# 849. Impact of a Combined Education and Data Driven Intervention on PrEP Uptake at the Veterans Health Administration

**DOI:** 10.1093/ofid/ofab466.1044

**Published:** 2021-12-04

**Authors:** Lewis Musoke, Kristen A Allen, Kaylee Bray, Erin J Lea, Janet Briggs, Amy H Shumaker, Brigid Wilson, Nicholas J Newman, Puja Van Epps

**Affiliations:** 1 VA Northeast Ohio Healthcare System; Case Western Reserve University, Sandusky, Ohio; 2 VA Northeast Ohio Health Care System, Westlake, Ohio; 3 VA Northeast Ohio Healthcare System, Avon Lake, Ohio; 4 Louis Stokes Cleveland VA Medical Center, Cleveland, OH; 5 VA NorthEast Ohio HealthCare System; Case Western Reserve University, Cleveland, Ohio

## Abstract

**Background:**

Despite proven efficacy, uptake of pre-exposure prophylaxis (PrEP) for HIV prevention in the US remains suboptimal. Whether electronic medical record (EMR) driven data tools increase PrEP uptake is unknown. Our study sought to understand the impact of education and an EMR data tool to increase PrEP uptake at the Veterans Northeast Ohio Healthcare System (VANEOHS).

**Methods:**

Using EMR data we identified persons at the VANEOHS with a diagnosis of bacterial Sexually Transmitted Illness (STI) as defined by a positive syphilis, gonorrhea or chlamydia test in the past 6 months. Beginning October 2020 Infectious Diseases (ID) staff launched an intensive PrEP education campaign for Primary care providers (PCP) and the emergency room (ER). During a 6-week intervention period, a ‘PrEP candidacy’ note was placed for the PCP in selected patients’ charts with recommendations for PrEP initiation and STI co-testing if appropriate. We measured the impact of the intervention on PrEP initiations from 3/1/21-5/31/21 and compared it to a pre-intervention period of 7/1/20-9/30/20 when candidates were identified in primary care only. We extracted pertinent data through the EMR and presented descriptive statistics as means and percentages. We compared outcomes using Chi-square test with simulated p-values due to small expected values.

**Results:**

Forty-two potential PrEP candidates were identified during post-intervention period compared to 6 in the pre-intervention period. The post-intervention candidates included cis-gender women (5/42, 12%) and ER referrals (6/42, 14%), both absent from the pre-intervention cohort. Compared to the pre-intervention period there was an increase in PrEP consults to ID (6 vs. 16; p=0.003) and PrEP starts (4 vs. 9; p=0.04). We observed increased rates of STI (69% vs. 50%) and HIV co-testing (79% vs. 67%) from pre to post intervention but these were not statistically different. Of the 42 candidates, 24 had been identified using the STI data tool. Of these, only 4 were referred for PrEP and none were initiated on PrEP by the end of our observation period.

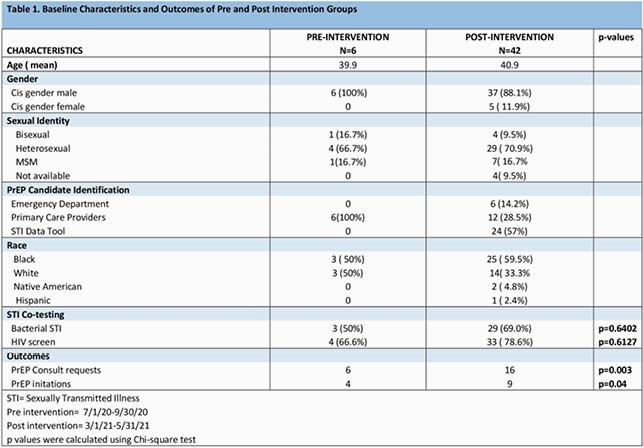

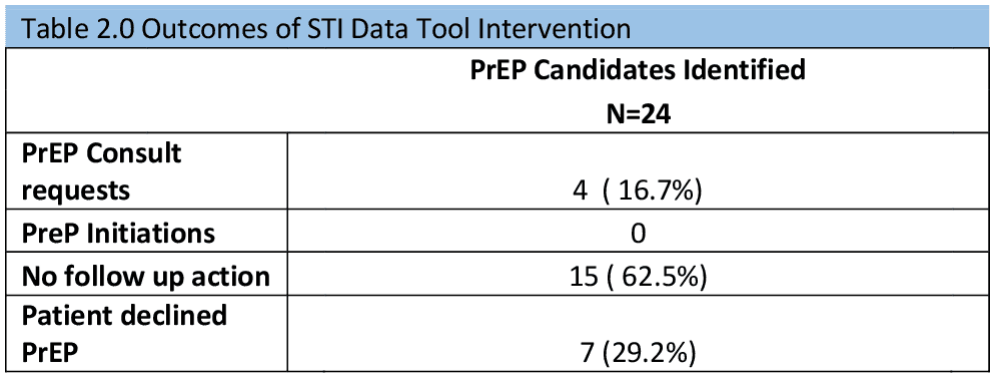

**Conclusion:**

The use of the data tool had no direct impact on PrEP uptake. Instead, the doubling of PrEP starts was attributable to education. Further studies are needed to maximize the utility of data tools to increase PrEP uptake.

**Disclosures:**

**All Authors**: No reported disclosures

